# Beta-Lactams Dosing in Critically Ill Patients with Gram-Negative Bacterial Infections: A PK/PD Approach

**DOI:** 10.3390/antibiotics10101154

**Published:** 2021-09-24

**Authors:** Kelly L. Maguigan, Mohammad H. Al-Shaer, Charles A. Peloquin

**Affiliations:** 1 Pharmacy Department, University of Florida Health Shands Hospital, Gainesville, FL 32608, USA; maguik@shands.ufl.edu; 2 Infectious Disease Pharmacokinetics Lab, College of Pharmacy and Emerging Pathogens Institute, University of Florida, Gainesville, FL 32610, USA; peloquin@cop.ufl.edu

**Keywords:** beta-lactams, PK/PD, critical care, resistance, Gram-negative

## Abstract

Beta-lactam antibiotics are often the backbone of treatment for Gram-negative infections in the critically ill. Beta-lactams exhibit time-dependent killing, and their efficacy depends on the percentage of dosing interval that the concentration remains above the minimum inhibitory concentration. The Gram-negative resistance rates of pathogens are increasing in the intensive care unit (ICU), and critically ill patients often possess physiology that makes dosing more challenging. The volume of distribution is usually increased, and drug clearance is variable. Augmented renal clearance and hypermetabolic states increase the clearance of beta-lactams, while acute kidney injury reduces the clearance. To overcome the factors affecting ICU patients and decreasing susceptibilities, dosing strategies involving higher doses, and extended or continuous infusions may be required. In this review, we specifically examined pharmacokinetic models in ICU patients, to determine the desired beta-lactam regimens for clinical breakpoints of *Enterobacterales* and *Pseudomonas aeruginosa,* as determined by the European Committee on Antimicrobial Susceptibility Testing. The beta-lactams evaluated included penicillins, cephalosporins, carbapenems, and monobactams. We found that when treating less-susceptible pathogens, especially *P. aeruginosa*, continuous infusions are frequently needed to achieve the desired pharmacokinetic/pharmacodynamic targets. More studies are needed to determine optimal dosing strategies in the novel beta-lactams.

## 1. Introduction

Antimicrobial resistance is a growing concern around the world, and it is estimated that by 2050, antimicrobial resistance may be responsible for up to 10 million deaths per year [1]. The increasing rates of Gram-negative resistance are especially alarming in extended-spectrum beta-lactamase (ESBL) *Enterobacterales*, carbapenem-resistant *Enterobacterales*, and multi-drug-resistant (MDR) *Pseudomonas aeruginosa* and *Acinetobacter* [2]. In critically ill patients, Gram-negative sepsis is a leading cause of morbidity and mortality [3]. MDR Gram-negative pathogens, and inappropriate empiric and definitive therapy have been identified as risk factors for mortality in intensive care unit (ICU) patients [4,5]. 

Beta-lactams make up the antibiotic backbone for the treatment of Gram-negative sepsis in critically ill patients. These agents are a first-line recommendation for frequently encountered infections, such as pneumonia, bacteremia, intra-abdominal infections, endocarditis, and urinary tract infections. Four specific classes of beta-lactams are used in clinical practice, including penicillins, cephalosporins, carbapenems, and monobactams. All beta-lactam classes consist of a four-member ring, referred to as the beta-lactam ring or azetidinone [6]. Monobactams are monocyclic, while the remaining classes are fused to a five- or six-member ring. Beta-lactams will covalently bond to penicillin-binding proteins (PBP) that will affect the production of peptidoglycans and bacterial cell wall synthesis [7]. Over time, resistance mechanisms have developed against the beta-lactam backbone, which has affected the overall efficacy of these agents. Major mechanisms of resistance include the production of beta-lactamases, reduced binding to PBP, overproduction of PBP, expression of transmembrane efflux pumps, and loss of outer membrane porins [8]. In combination with over-prescribing, increasing rates and mechanisms of resistance are rendering the beta-lactams less effective, thus the optimization of pharmacokinetic/pharmacodynamic (PK/PD) properties is of the upmost importance.

Critical illness can significantly alter the PK/PD properties of beta-lactams, and under-estimating the effects of these changes can lead to clinical failure. Changes in volume of distribution (Vd) and clearance are the most common factors affecting beta-lactam PK/PD, and changes in dose and infusion time are often necessary [9]. Fluid resuscitation, hypoalbuminemia, renal-replacement therapy (RRT), and extracorporeal membrane oxygenation (ECMO) can increase Vd. Clearance is increased in hypermetabolic states and augmented renal clearance (ARC), and decreased in patients with acute kidney injury (AKI) [9,10]. 

Between the increasing rates of Gram-negative resistance and altered PK in the critically ill, it is crucial to select the correct dose, interval, and infusion duration. Beta-lactams exhibit time-dependent killing, and in ICU patients, it may be beneficial to target a free drug concentration that is 100% above the minimum inhibitory concentration (100% fT > MIC), and concentrations that are four times the MIC (100% fT > 4 × MIC), compared to 40–70% fT > MIC in non-ICU patients [11,12,13]. Achieving these desired PK/PD targets is associated with microbiological success, lower rates of clinical failure, and improved survival [13,14,15,16]. In addition, prolonging beta-lactam infusion may optimize the PK/PD target, and was successful in suppressing the emergence of resistance in some pre-clinical studies [17,18,19].

Therapeutic drug monitoring (TDM) is generally recommended in this population; however, it is not routinely available at most institutions [11]. Thus, when determining empiric beta-lactam regimens in critically ill patients with less-susceptible pathogens, it is often necessary to consult published PK models. In this review, we evaluated the literature related to the PK/PD of beta-lactams and assessed the dosing for Gram-negative bacterial infections, based on the breakpoints reported by the European Committee on Antimicrobial Susceptibility Testing (EUCAST).

## 2. Literature Review

A comprehensive search of the PubMed and Medline databases was conducted to identify relevant articles to include in the review. We used the search terms ‘beta-lactams’, ‘pharmacokinetics’, ‘pharmacodynamics’, and ‘critical care’. All the ICU populations were included in the search, with the most common populations being medical and surgical ICU patients. We targeted articles that included MIC values of *Enterobacterales* and *P. aeruginosa* that were less susceptible and closer to the established EUCAST resistant breakpoint. The EUCAST breakpoints for the beta-lactams evaluated are provided in Table 1.

### 2.1. Penicillins

Piperacillin/tazobactam (TZP) is the only commercially available antipseudomonal penicillin in the United States. Even though standard intermittent dosing is recommended in the package insert, a dosing strategy of 4.5 g q6 h has demonstrated a decreased likelihood to achieve adequate exposure in critically ill patients when targeting an MIC of 16 mg/L [20]. Utilizing extended-infusion (EI) TZP was thought to overcome the limitations of intermittent dosing strategies. EIs have demonstrated an increased probability of achieving 100% fT > MIC, as well as improved clinical outcomes, such as increased 30-day survival and clinical cure [21]. However, in critically ill patients with normal or augmented renal function, EI still resulted in an inability to achieve the recommended PK/PD targets [22]. 

Continuous infusion (CI) TZP may be advantageous in critically ill patients with ARC, or infections with higher MICs. Roberts, et al. evaluated first-dose and steady-state PK in 16 critically ill patients with normal renal function, who received either 16 g/day by intermittent bolus or a 4 g initial bolus followed by 8 g/day CI on day 1 and 12 g/day starting on day 2. Five blood samples were obtained during the initial bolus dose, twelve samples after the bolus dose, and ten steady-state samples were drawn at the start of day 2. Intermittent, EI, and CI dosing strategies were simulated from the developed population PK model. The probability of target attainment (PTA) was defined as free piperacillin concentrations greater than the MIC for 50% of the dosing interval. The PTA was only 79% and 59% for an MIC of 0.25 mg/L for the 4 g bolus dose, administered q6 h and q8 h, respectively. However, the 12 g and 16 g CI was able to achieve 100% PTA for MICs of 4 and 8 mg/L. Only the 16 g CI would achieve 66% PTA when the MIC was 16 mg/L, suggesting that even higher doses would be necessary for more resistant pathogens [23]. 

Two similar studies were conducted in the early phase of sepsis and septic shock, but included patients with impaired renal function, not requiring renal replacement therapy [24,25]. Obrink-Hansen, et al. evaluated piperacillin 4 g q8 h, administered over 3 min in 15 patients with septic shock [24]. Eight blood samples were obtained after the third dose and a PK model was developed to assess empiric dosing. The investigators targeted the MIC breakpoint for *P. aeruginosa* (16 mg/L), and the PK/PD target chosen was 50% fT > 4 × MIC and 100% fT > MIC. Increased serum creatinine was associated with decreased piperacillin clearance (*p* = 0.005), thus patients with impaired renal function were more likely to achieve the desired PK/PD targets. However, with an MIC of 16 mg/L, standard intermittent dosing (4 g q8 h) was unable to achieve a PTA > 90%, even when the serum creatinine was 250 μmol/L. Further, 12 and 16 g/day CI was able to achieve 100% fT > MIC and 50% fT > 4 × MIC of 8 mg/L, but 16 g/day CI was the only regimen found to achieve both 100% fT > MIC and 50% fT > 4 × MIC of 16 mg/L [24]. Sukarnjanaset, et al. demonstrated similar results, and developed a PK model from 48 critically ill patients with early sepsis and varying severities of renal impairment. Lower creatinine clearance (CrCl) and mean arterial pressure (MAP) were associated with decreased piperacillin clearance. Standard intermittent dosing strategies also did not achieve 90% fT > MIC for pathogens with an MIC of 16 mg/L, in patients with CrCl > 60 mL/min; however, 4 g administered over 2 h q6 h was able to achieve 90% fT > MIC. When considering the cumulative fraction response (CFR) for *Klebsiella pneumoniae*, CI regimens were more effective at achieving a 90% CFR in patients with normal renal function. However, none of the regimens evaluated were able to achieve 90% CFR for *P. aeruginosa* [25].

The optimal dose of CI piperacillin was evaluated by Dhaese and colleagues in 110 surgical ICU patients who were not receiving RRT or ECMO [26]. The patients received a 4 g loading dose prior to being initiated on CI, which was dosed based on CrCl (<15 mL/min: 8 g/day; 15–29 mL/min: 12 g/day; 30–129 mL/min: 16 g/day; 130–199 mL/min: 20 g/day; >200 mL/min: 24 g/day). The investigators targeted 100% fT > 4 × MIC and the dosing was considered successful if the fractional target attainment (TA) was >95%. One hundred percent TA with MIC = 8 mg/L was achieved for >12 g/day CI in patients with CrCl >60 mL/min. However, 100% TA could not be achieved with MIC = 16 mg/L in the 24 g/day CI cohort when CrCl exceeded 90 mL/min, suggesting that alternative antibiotic strategies may be necessary in patients with ARC [26]. 

Utilizing CI TZP also results in a greater TA of the beta-lactamase inhibitor tazobactam, compared to intermittent dosing strategies. The target tazobactam levels depend on the levels of beta-lactamase expression. For strains expressing higher levels of beta-lactamase, TA was defined as concentrations above 2 mg/L free tazobactam for 85% of the dosing interval. A model was created using data from 18 critically ill patients, and demonstrated that a TZP 16/2 g CI/day would result in >75% TA for tazobactam [27]. Further, 16/2 g CI/day would also result in adequate alveolar concentrations of piperacillin and tazobactam [28]. Utilizing this daily dose resulted in alveolar concentrations exceeding a breakpoint of 16 mg/L for the majority of patients receiving 16/2 g CI/day. A total daily dose of 12/1.5 g CI/day resulted in less TA [28].

### 2.2. Cephalosporins

The early work on cefepime suggested that CI and more frequent administration would be needed in critically ill patients, to achieve the optimal TA [29]. Tam et al. simulated different cefepime dosing regimens using a population PK model, with patients having different conditions (healthy, renal insufficiency, liver impairment, and cystic fibrosis), and used different CrCl values (60, 90, and 120 mL/min). The PK/PD target chosen was 83% fT > 4.3 × MIC. Cefepime 2 g q12 h (6-h infusion), 2 g q8 h (30-min infusion), and 4 g CI achieved >80% TA at MIC 4 mg/L for CrCl 60 mL/min. Cefepime 4 g CI was needed to achieve good TA at MIC 4 mg/L (any CrCl value) and 8 mg/L (CrCl 60 mL/min). The TA dropped significantly at higher MICs and higher CrCl values [30]. Roos et al. developed a cefepime population PK model using data from 13 ICU patients. Twelve blood samples were drawn from each patient at the following two occasions: after the first dose and at steady state (day 3–6). For a target of 65% fT > MIC, 2 g q8 h and 1 g q4 h intermittent infusions achieved >80% TA at MIC 4 mg/L, but did not do as well with higher MICs; whereas, 0.5 g loading, followed by 2–6 g CI, achieved good TA at MIC 4 mg/L, 4–6 g CI at MIC 8 mg/L, and 6 g CI at MIC 16 mg/L [31]. Another PK model was developed, using cefepime concentrations from 26 ICU patients who had ventilator-associated pneumonia (VAP). The model was validated in another set of six patients. The authors simulated cefepime 1–2 g q8–12 h, infused over 30 min and 3 h regimens, and used CrCl values from 10 to 120 mL/min. Using a PK/PD target of 50% fT > MIC, all the regimens achieved TA >80% at CrCl values below 50 mL/min and MICs ≤ 8 mg/L. At higher CrCl, 2 g q8 h achieved the best TA at MIC’s 4 mg/L and 8 mg/L, with the regimens infused over 3 h being superior to 30-min infusion [32]. A recent cefepime PK model was developed, including 266 ICU patients, which simulated different cefepime regimens, and assessed the attainment of the targets 100% fT > MIC and fT > 4 × MIC. For the target 100% fT > MIC, 2 g q6 to q8 h EI will be needed at CrCl values below 90 mL/min, and MICs 4 mg/L and 8 mg/L. At higher CrCl (>90 mL/min) or MIC 16 mg/L, a 4 g loading dose, followed by 7–8 g CI, will be needed. For 100% fT > 4 × MIC, only the 7–8 g CI regimens achieved TA of around 80% at MIC 4 mg/L, but not at 8 mg/L [33]. In two studies, which included neurocritically ill patients, cefepime showed better TA, both in the plasma and cerebrospinal fluid (CSF), when administered as a CI or q8 h, compared to q12 h at MICs 4 mg/L and 8 mg/L. Additionally, patients treated with CI had a shorter therapy duration [34,35]. Similarly, a 4 g CI cefepime regimen showed good penetration to the epithelial lining fluid (ELF) (~100%), while intermittent infusion achieved an undetectable sputum concentration [36,37].

Georges and colleagues developed a ceftazidime population PK model using data from 49 ICU patients for model building and 23 for model validation. The authors found a correlation between ceftazidime clearance and glomerular filtration rate (GFR), the central Vd with mechanical ventilation, and the peripheral Vd with the reason for admission. Simulating both intermittent and CI regimens, the authors showed that ceftazidime 6 g CI achieved a better TA compared to intermittent infusion at a PK/PD target up to 100% fT > 5 × MIC [38]. Patients with high CrCl may need higher doses [39]. A retrospective study investigated the PK of ceftazidime CI in 92 ICU patients. The dose range was from 1 g to 6 g per day, and the mean CrCl was 94 mL/min (range, 14–258 mL/min). The mean ceftazidime concentration was 46.9 mg/L (range, 7.4–162.3 mg/L), and the 100% T > 5 × MIC target was achieved in 84% of the 51 patients who had confirmed infection. No adverse events were reported [40]. Stein et al. evaluated the PK of ceftazidime/avibactam in ten ICU patients. Plasma samples were drawn at 2, 4, 6, and 8 h after the patients received multiple doses of the drug. The TA for 50% fT > MIC for ceftazidime and 50% fT > 1 mg/L for avibactam was more than 90% using a 2.5 g q8 h (2-h infusion) regimen at MICs up to 16 mg/L [41]. This regimen has shown high TA across different indications, using population PK models and data from phase 3 trials [42]. In patients with severe intra-abdominal infections, ceftazidime CI showed superior target attainment in both serum and peritoneal exudate, compared to intermittent infusion (T > 4 × MIC > 90% vs. 44%, respectively) [43]. Similarly, the concentration of ceftazidime was higher in ELF when administered as CI, and achieved better TA [44,45].

In studies investigating the PK of unbound ceftriaxone in patients, ceftriaxone 2 g/day CI achieved excellent TA at MICs up to 2 mg/L for the target 100% fT > MIC, and 2 g q12 h for the target 50% fT >MIC. In these population PK models, CrCl was associated with the clearance of unbound ceftriaxone, which affects the TA [46,47]. One study found that ARC increased the probability of failure to achieve the desirable PK/PD target in patients receiving ceftriaxone, and suggested that ceftriaxone 2 g q12 h would be needed for optimal TA in patients with CrCl > 200 mL/min [48]. A PK model was developed using plasma and CSF samples from patients with bacterial meningitis. A nomogram for twice-daily dosing was developed accordingly, using the estimated GFR (eGFR) and the body weight to help target ceftriaxone plasma concentrations between 20 mg/L and 100 mg/L. The daily dose ranged from 20 to 160 mg/kg/day, and the eGFR from 15 to 155 mL/min/1.73 m^2^ [49]. Patients who received high-dose ceftriaxone, a median daily dose of 6.5 g (range, 4–9 g), which corresponds to a median of 97.5 mg/kg (range, 77–131 mg/kg), achieved a median total CSF concentration of 13.3 mg/L (range, 0.9–91.2 mg/L) [50].

Sime et al. developed a population PK model using an unbound ceftolozane/tazobactam concentration from critically ill patients. The authors enrolled 12 patients prospectively. The clearance of drugs was correlated with the urinary CrCl, and the Vd was related to the weight. Simulating multiple dosing regimens at different CrCl, and evaluating TA at 40%, 60%, and 100% fT > MIC for ceftolozane and 20% fT > 1 mg/L for tazobactam, ceftolozane/tazobactam 1.5 g q8 h, might be sufficient to achieve good TA at CrCl values ≤140 mL/min/1.73 m^2^; however, 1.5 g loading, followed by 4.5 g CI, will be needed if a higher MIC is suspected and/or the patient has ARC [51]. Another prospective study evaluated the TA of ceftolozane/tazobactam intermittent, extended, and CI for different PK/PD targets and MICs. The regimen 2 g q8 h, infused over 4 h or as CI, achieved good TA at MICs up to 16 mg/L for the PK/PD target 100% fT > MIC. Whereas, for 100% fT > 4 × MIC, only 6 g CI achieved TA >90% at an MIC up to 8 mg/L [52]. The penetration of ceftolozane/tazobactam penetration to the CSF was evaluated and the mean (SD) CSF:plasma ratio was 0.2 (0.2). The TA in the CSF was poor with 3 g q8 h and 9 g CI regimens [53]. The penetration to the ELF was ~50% in patients who received 3 g q8 h, or was adjusted for renal function. The mean ceftolozane and tazobactam concentration remained above 4 mg/L and 1 mg/L, respectively, for 100% of the dosing interval [54].

### 2.3. Carbapenems

Prolonged meropenem infusions are associated with lower mortality and increased clinical cure in critically ill patients [21,55]. Compared to non-critically ill patients, ICU patients often have lower meropenem TA rates, especially in isolates with MICs of 4 and 8 mg/L, and prolonged infusions are often necessary to achieve the target concentrations [56]. Early PK models suggest that it may be necessary to utilize EI or CI meropenem in patients with normal renal function, to achieve a CFR and higher plasma concentrations. Roberts, et al. developed a model from 10 critically ill patients with sepsis and normal renal function, utilizing 15 day 1 and 9 steady-state concentrations on days 2–5. PTA was defined as 40% fT > MIC and dosing regimens were considered successful if the CFR was 100%. The investigators found that meropenem clearance was dependent on renal function, and it was difficult to achieve PK/PD targets in isolates with higher MICs. Utilizing CI with 3 or 6 g/day, or EI with 2 g q8 h over 4 h was able to achieve 100% and 96.9% CFR, respectively, when the *P. aeruginosa* MIC was 8 mg/L [57]. Minichmayr, et al. developed a dosing nomogram for CI meropenem in critically ill patients with varying severities of renal dysfunction. Steady-state blood samples were collected from 195 ICU patients. The patients received 0.5–6 g/day and all the concentrations obtained exceeded 2 mg/L. Further, 99.8% and 90.3% of the concentrations exceeded 4 mg/L and 8 mg/L, respectively. The nomogram that was developed used the Cockcroft-Gault formula and the desired target concentration to estimate the daily dose required. To target a concentration of 16 mg/L, the daily dose can be estimated using the following equation: 0.0378 ×CrCl + 1.07 [58]. 

Increased total daily doses of meropenem may be necessary to achieve 50% fT > MIC in ELF [59]. Benitez-Cano, et al. evaluated intrapulmonary concentrations of CI meropenem. The investigators found that a 2 g loading dose, followed by 3 g/day CI, only achieved the optimal TA for organisms with an MIC of <2 mg/L. However, PTA ≥ 90% was achieved with a 6 g/day CI for organisms with an MIC up to 2 mg/L [59]. Thus, a higher daily dose of meropenem CI may be warranted in severe lung infections. 

Imipenem is used in combination with the renal dehydropeptidase inhibitor cilastatin. The IMPACT study evaluated imipenem administered q8 h over 30 min in 51 ICU patients with VAP, using six steady-state samples around the fourth dose, to evaluate dosing strategies using a population approach. PTA was defined as 40% fT > MIC, and EUCAST breakpoints of 2 and 4 mg/L were specifically evaluated. Further, 99.1% (0.75 g q6 h) and 99.4% (1 g q6 h) of simulated patients achieved the TA for MIC = 2 mg/L. PTA for MIC = 4 mg/L was lower, at 33% and 45%, respectively, but higher than the identical dose regimens with q8 h intervals (14% and 21%, respectively) [60]. Chen, et al. demonstrated similar results and developed a population model from 247 ICU patients with 580 plasma imipenem levels. A regimen of 0.75 g q6 h reached the treatment targets of 40% fT > MIC in 99.5% and 96.5% of simulated patients with an MIC of 2 mg/L and 4 mg/L, respectively. Further, 70% fT > MIC was 90.8% and 67.8%, respectively. The authors did suggest that 1 g q6 h may be necessary in patients with more resistant pathogens [61]. Finally, Jaruratanasirikul, et al. evaluated the PTA of imipenem regimens at various GFRs in 50 ICU patients. For 60–120 mL/min, 4-h infusions were necessary to achieve 90% PTA of 75% fT >MIC. A 0.5 g q6 h dosing scheme for MIC = 2 mg/L and 1 g q6 h for MIC = 4 mg/L was required. In GFR 30–59 mL/min, 0.5 g q8 h for a 4-h infusion, or 0.5 g q6 h for a 1-h infusion reached at least 90% PTA for an MIC = 2 mg/L. A regimen of 1 g q6 h for a 1-h infusion or 1 g q8 h for a 1-h infusion was needed for an MIC = 4 mg/L. When GFR is 15–29.9 mL/min, 0.5 g q8 h (1- or 4-h infusion) should be used for an MIC = 2 mg/L, and 0.5 g q6 h (1- or 4-h infusion) should be used for an MIC = 4 mg/L [62].

Roberts, et al. conducted the first study evaluating doripenem PK/PD parameters in 31 critically ill patients with nosocomial pneumonia, receiving 250 mg or 500 mg as a 30-min, 1-h, or 4-h infusion. Steady-state troughs and 5–6 samples throughout the dosing interval were obtained. The targets assessed were 40% and 90% fT >MIC. A regimen of 500 mg q8 h as a 1- or 4-h infusion achieved the desired PK/PD targets when CrCl = 100 mL/min for organisms with MIC <2 mg/L. In patients with ARC (CrCl = 150 mL/min), the infusion should be extended to 4 h [63]. Jaruratanasirikul and colleagues found that higher doses may be needed to achieve 40% and 80% fT > MIC. Further, 93% and 98% PTA for achieving 40% T > MIC was observed in the 1 g q8 h regimens administered over 1 and 4 h, respectively. The 2 g q8 h over 4 h was the only regimen that resulted in >90% PTA for 80% T > MIC [64]. Utilizing a 4 h infusion will also result in higher ELF concentrations compared to a 1 h infusion [65]. Oesterreicher, et al. found that a 4 h infusion of 1 g resulted in a maximum concentration of 6.9 mg/L, compared to 4.6 mg/L for the 1 h infusion [65].

Ertapenem exerts no activity against *P. aeruginosa*, but can be used to treat *Enterobacterales*. Adequate plasma and ELF concentrations exceeding MIC_90_ values have been observed in patients with VAP, receiving a standard dose of ertapenem of 1 g q24 h; however, only a few PK studies have been performed in ICU patients [66]. Burkhardt, et al. used free drug concentrations in 17 critically ill patients with VAP, receiving 1 g q24 h, to develop a PK model. The patients who were enrolled were observed to have increased Vd and clearance compared to healthy controls, which resulted in a lower Cmax and area under concentration–time curve. In the model, the plasma concentrations were 2 mg/L for 6 h of the dosing interval, which was likely related to the low serum albumin levels observed in the population. The investigators suggested that for critically ill patients with hypoalbuminemia, the dosing interval may need to be shortened or changed to a continuous infusion; however, there are limited data on the stability of ertapenem when it is administered as a CI [67]. Liebchen, et al. demonstrated conflicting results in six ICU patients with hypoalbuminemia. The standard dose of ertapenem (1 g q24 h) exceeded 2 mg/L and 0.25 mg/L for 72% and 100% of the dosing interval, respectively, suggesting that additional studies are still warranted [68]. 

### 2.4. Monobactam

Aztreonam is a monobactam that is frequently used empirically against aerobic Gram-negative bacteria in patients with a documented history of immunoglobulin E-mediated anaphylaxis. Aztreonam also has an evolving role in the treatment of resistant Gram-negative pathogens, such as metallo-β-lactamase (MBL)-producing Enterobacteriaceae [69]. The recommended dosing regimen is 2 g q6-8 h in ICU patients with normal renal function, but the dose should be halved when CrCl is 10–30 mL/min/1.73 m^2^ and quartered when CrCl is <10 mL/min/1.73 m^2^ [70,71]. 

Cornwell, et al. evaluated aztreonam PK in 30 critically ill, surgical ICU patients who were receiving 2 g q6 h (30-min infusion). The investigators obtained trough blood samples and samples at 30 min, 2.5 h, and 5 h after the infusion. The target concentration was ≥8 mg/L; however, the MICs of isolated organisms were not reported. The patients were primarily young, male adults with respiratory or intraabdominal infections. Despite an observed mean Vd of 0.35 L/kg, which is substantially higher than the estimated Vd of 0.18 L/kg in healthy volunteers, 68% of the patients achieved aztreonam concentrations ≥ 8 mg/L for the entire dosing interval and 89% of aztreonam concentrations obtained for all the patients were ≥ 8 mg/L. In the nine patients who did not achieve a trough concentration ≥ 8 mg/L for the entire dosing interval, eight had documented clinical cure. Thus, despite the increased Vd observed, aztreonam 2 g q6 h (30-min infusion) achieved adequate TA in a young critically ill surgical population [72]. 

McKindley, et al. also evaluated the pharmacokinetic profile of aztreonam in critically ill adult trauma patients who were mechanically ventilated, being treated for pneumonia. The patients received aztreonam 2 g q6 h (30-min infusion), and blood samples were obtained at 0.5, 1, 2, 4, and 7 h after the infusion. To evaluate pulmonary disposition of aztreonam, sputum samples were obtained two hours after the end of the infusion. Nine patients, with an average age of 51 years, with normal renal function were included in the study. The investigators demonstrated significantly increased Vd compared to healthy controls, at 2–3 days and 7–8 days (0.42 and 0.31 vs. 0.21 L/kg, *p* < 0.05), and a prolonged half-life (3.9 and 2.6 h vs. 1.7 h, *p* < 0.05). There was no observed difference in clearance compared to the controls. The CrCl estimates and total clearance did demonstrate good association with aztreonam at 2–3 days (r^2^ = 0.73). The average sputum concentration at 2–3 days was 5.9 mg/L and one sample at 7–8 days was 9.7 mg/L, but these results could not be compared, due to a lack of sampling in the 7–8-day period [73]. Similar to Cornwell, et al., the investigators demonstrated an increased Vd in critically ill patients, suggesting that 2 g q8 h may be inadequate in critically ill trauma patients. [72,73]. 

Falcone and colleagues developed a PK model in adult patients with documented carbapenemase producing *Enterobacterales,* who received combination therapy with aztreonam and ceftazidime/avibactam. Forty-one patients were included in the model and 20 patients were admitted to the ICU at the time of analysis. The median age was 75 years old, with a median body mass index (BMI) of 23.9 kg/m^2^. The patients received 1–2 g q8 h (administered over 2 h), and blood samples were obtained prior to the first, fourth and fifth dose, at the end of the infusion, and around the midpoint of the dosing interval. The lowest simulated dose to achieve 90% PTA with an MIC of 4 mg/L was 1 g q8 h, when eGFR was 15–120 mL/min, and 2 g q8 h when eGFR was >120 mL/min. For an MIC of 8mg/L, aztreonam 1 g q8 h for eGFR 15–60 mL/min, 2 g q8 h for eGFR 60–90 mL/min, 2 g q6 h for eGFR 90–120 mL/min, and 2 g loading dose, followed by 8 g CI/day, achieved 90% PTA. For an MIC of 16 mg/L, 90% PTA was only achievable when eGFR was <90 mL/min. Aztreonam 1 g q8 h was required for eGFR 15–30 mL/min, 2 g q6 h for eGFR 30–60 mL/min, and 2 g loading dose, followed by 8 g CI/day, for eGFR 60–90 mL/min [74].

Table 2 summarizes the initial dosing recommendation for beta-lactams used to treat resistant Gram-negative infections. The provided doses are for patients with normal organ function, and therapeutic drug monitoring should be utilized if available, to optimize the dosing regimen.

## 3. Stability

The physiochemical stability of prolonged and CI beta-lactams should be considered when selecting a therapeutic regimen. Improper consideration could lead to drug degradation over time and a loss of therapeutic efficacy. Antibiotic loss should not exceed more than 10% in a 24 h period, as established by the United States Pharmacopeia [75]. Stability varies amongst beta-lactams, but carbapenems exhibit the most instability, while piperacillin/tazobactam and aztreonam are the most stable [75,76]. The recommended administration times of continuous infusions are provided in Table 3 [75,77,78,79,80,81]. Because meropenem exhibits the most instability, infusions must be changed every twelve hours, to reduce the risk of drug degradation [80]. Few studies exist on continuous infusion of ertapenem, and the only study that has been published did not provide administration instructions [82]. Due to the short stability of ertapenem, the continuous infusion would likely need to be changed every six hours [83]. Further studies are needed to evaluate ertapenem stability when it is administered by continuous infusion.

## 4. Safety and Limitations

Targeting less-susceptible pathogens will require increased exposure to beta-lactam therapy, through increased total daily doses and prolonged infusions. Increased exposure introduces the possibility of toxicities, which include neurotoxicity, nephrotoxicity, hepatotoxicity, and genotoxic effects [84]. Relative pro-convulsive activity among the beta-lactams discussed is the highest in cefepime, imipenem, and aztreonam [84,85]. Nephrotoxicity is rare, but can present as acute interstitial nephritis (AIN), nephropathy, due to hemolytic anemia, and acute kidney injury. AIN more frequently occurs in penicillins and cephalosporins [84,86]. Ceftriaxone and piperacillin are more frequently implicated in hemolytic anemia, while piperacillin has the highest incidence of drug-induced AKI, especially when used in combination with vancomycin [84,87,88,89,90].

Unfortunately, therapeutic values associated with toxicity are not well established for all beta-lactams. However, it is suggested that the clinical threshold for toxicity is a trough of >44.5 mg/L, 20 mg/L, and 361 mg/L for meropenem, cefepime, and piperacillin alone, respectively [11,84]. When considering that providers may frequently target 100% fT > 4 × MIC, patients with pathogens with higher MICs may be at a higher risk for toxicity. For example, if targeting 100% fT > 4 × MIC for a pathogen with an MIC of 8 mg/L, being treated with cefepime, providers will aim to achieve a trough concentration of ~32 mg/L. This trough concentration is >20 mg/L, which may predispose the patient to possible drug toxicities. Thus, in patients with risk factors for beta-lactam toxicities and less-susceptible pathogens, it may be prudent to target alternative PK/PD targets, such as 100% fT > MIC [91].

Some limitations to this review that may impact clinical applicability include dosing in changing organ function and special populations. Critically ill patients experience frequent changes in renal function and it is challenging estimating renal dysfunction with predictive equations. It is estimated that AKI occurs in 20–50% of patients in the ICU, while rates of ARC may occur in up to 65% of patients [92,93]. Novel biomarkers may be more beneficial in estimating the severity of AKI, compared to traditional biomarkers, such as urine output and serum creatinine, but have not been utilized in PK studies so far [94]. This can make dosing challenging in patients with fluctuating renal function, and more frequent therapeutic drug monitoring will often be required. 

This review did not address dosing recommendations in populations that may warrant special considerations, such as obesity, ECMO, or RRT. Obesity can lead to lower beta-lactam exposure and TA compared to non-obese patients, while drug sequestration and protein binding can lead to variable drug concentrations in patients receiving ECMO and RRT [95,96]. A detailed review of the effects of dosing in these populations is beyond the scope of this narrative [95,97].

## 5. Conclusions

Less-susceptible Gram-negative pathogens in critically ill patients warrant initial beta-lactam dosing strategies that utilize extended or continuous infusions, to optimize TA, especially in patients with ARC. Patients with impaired renal impairment often still require extended infusions to achieve PK/PD targets. TDM should follow to individualize therapy. More studies are needed to evaluate the desired regimen in novel beta-lactam antibiotics. 

## Figures and Tables

**Table 1:** EUCAST MIC breakpoints ^1^.

Beta-Lactam	*Enterobacterales*		*Pseudomonas* spp.	
	Susceptible≤	Resistant>	Susceptible≤	Resistant>
Piperacillin/tazobactam	8	8	0.001	16
Cefepime	1	4	0.001	8
Ceftazidime	1	4	0.001	8
Ceftazidime/avibactam	8	8	8	8
Ceftolozane/tazobactam	2	2	4	4
Ceftriaxone	1	2	-	-
Meropenem	2	8	2	8
Imipenem/cilastatin	2	4	0.001	4
Doripenem	1	2	0.001	2
Ertapenem	0.5	0.5	-	-
Aztreonam	1	4	0.001	16

^1^ The European Committee on Antimicrobial Susceptibility Testing. Breakpoint tables for interpretation of MICs and zone diameters, version 11.0, 2021.

**Table 2:** Suggested beta-lactams’ initial dosing for resistant Gram-negative infections in patients with normal renal and hepatic function.

Beta-Lactam	Regimen
Piperacillin/tazobactam	16 g/day CI
Cefepime	6 g/day CI
Ceftazidime	6 g/day CI
Ceftazidime/avibactam	2.5 g q8 h (2-h infusion)
Ceftolozane/tazobactam	6 g/day CI
Ceftriaxone	2 g q12 h or 4 g CI ^1^
Meropenem	3–6 g/day CI or 2 g q8 h (4-h infusion)
Imipenem/cilastatin	1 g q6 h (4-h infusion)
Doripenem	1 g q8 h (4-h infusion)
Ertapenem	1 g/day q24 h (30-min infusion) ^2^
Aztreonam	2 g q6 h (2-h infusion) or 2 g load then 8 g/day CI

^1^ A dosing nomogram based on renal function and body weight is also available [49]. ^2^ Lack of consistent stability data with CI at this time.

**Table 3:** Established stability of continuous infusion beta-lactams [75,77,78,79,80,81].

Beta-Lactam Continuous Infusion	Stability
Piperacillin/tazobactam	16 g/day administered over 24 h
Cefepime	6 g/day administered over 24 h
Ceftazidime	6 g/day administered over 24 h
Ceftolozane/tazobactam	6 g/day administered over 24 h
Meropenem	1.5–3 g q12 h administered over 12 h (3–6 g/day)
Aztreonam	8 g/day administered over 24 h

## Data Availability

Available data are presented in the manuscript.
